# Attitudes of medical students towards men who have sex with men living with HIV: implications for social accountability

**DOI:** 10.5116/ijme.5f87.39c2

**Published:** 2020-10-23

**Authors:** Willy Dunbar, Colette Alcide, Christian Raccurt, Jean W. Pape, Yves Coppieters

**Affiliations:** 1 Health Systems and Policies, International Health, School of Public Health, Université libre de Bruxelles (ULB), Brussels, Belgium; 2 Faculty of Health Sciences, Quisqueya University, Port-au-Prince, Haiti; 3 Haitian Study Group for Kaposi's Sarcoma and Opportunistic Infections (GHESKIO), Port-au-Prince, Haiti

**Keywords:** Haiti, HIV, men who have sex with men, medical student, social accountability

## Abstract

**Objectives:**

To explore the attitudes that medical students in Haiti harbour toward Men who have Sex with Men living with HIV in order to better understand how stigma and other factors may impair healthcare, and to explore suggestions of opportunities in line with the values of social accountability.

**Methods:**

This study employed a qualitative design by using a grounded theory approach regarding the context of Haiti. We used purposive sampling to select the 22 research participants. In-depth interviews were conducted, audio-recorded, transcribed and analyzed using an inductive content analysis approach.

**Results:**

Although stigmatizing attitudes emerged through the findings, medical students expressed willingness to provide Men who have Sex with Men with adequate health services in relation to HIV care. Their expressions were based on the Men who have Sex with Men's comprehensive right to receive equitable care, the moral responsibility of healthcare professionals, their perception of health disparities and the HIV global risk reduction. Participants pointed out that the medical education curriculum did not consider sexual health and specificities of sexual minorities and suggested a more inclusive and socially accountable training based on equity and quality.

**Conclusions:**

The students expressed favourable attitudes regarding health services to Men who have Sex with Men even though some layered stigmatizing attitudes emerged through the discussions. They all lacked skills on how to handle health specificities of sexual minorities. These findings recommend a revision of the medical education curriculum in regard to social accountability principles.

## Introduction

Advances in scientific understanding of HIV prevention and treatment by the global health community, government and civil society organizations have made it possible to control the epidemic.^1^ To achieve the goal of ending HIV by 2030, targets have been set by the United Nations for HIV diagnosis and care continuum. The 95-95-95 goals aim for 95% of individuals infected with HIV to be aware of their status, 95% of those diagnosed to initiate antiretroviral (ARV) treatment, and 95% of those on ARVs to have undetectable viral loads.^2^ While this goal has been set, in 2018, key populations (KP) and their sexual partners accounted for 54% of new HIV infections globally, and among the KP, Men who have Sex with Men (MSM) accounted for 17%.^3^ In Haiti, a country of the Caribbean, which is the most affected region after Africa, prevention and treatment strategies implemented in collaboration with international organizations and donors have contributed to reducing the national HIV prevalence from 6.2% in 1993 to 2% in 2018. However, according to a survey conducted and published by the United Nations, the HIV prevalence among MSM was 18.2% in 2017, making them the most underserved population in terms of HIV prevention and care.^4^^-^^6^

In order to fulfil the targets to properly include and retain MSM in the continuum of care, attitudes of caregivers play a critical role. As MSM are at an increased risk for HIV and continue to bear a disproportional burden of the infection, many barriers prevent or limit their access to proper care.^7^ Among the barriers to accessing healthcare, stigma, discrimination and confidentiality remain major concerns.^8^ Indeed, despite many efforts that are being made to improve the management of HIV, stigma remains a challenging issue.^9^ Therefore, as the response to the epidemic continues to grow, stigma continues to hamper the healthcare services, and people living with HIV (PLHIV) are often perceived as having socially despised behaviours.^10^^-^^12^

Previous studies conducted in several settings have reported suboptimal uptake of services among MSM; these findings have been attributed to a lack of trust and non-responsiveness to the health needs of MSM on the part of the providers.^8^^,^^13^ HIV sometimes evoke irrational emotions and fears, and the degree of bias perceived by patients in healthcare settings depends on the provider's knowledge about HIV, experience working with PLHIV and HIV-related fears.^14^^,^^15^ Medical students represent the next generation of clinicians responsible for HIV care efforts, and they often reflect attitudes held in their society and community of practice.^16^ Studies assessing attitudes of medical students towards MSM have also shown expression of discomfort with the behaviour of gay men and the prevalence of reluctance to provide proper medical care.^17^^,^^18^ As MSM are subjected to layers of bias, their perception of negative attitudes is associated with reduced enrolment, reduced treatment adherence and poor medical outcomes.^19^^,^^20^ In this regard, medical education and services should be directed towards eliminating the concerns and health priorities of the society and take into account the complexity and changing expectations of health systems, hence the implications of the social accountability concept advocating for equity, quality, relevance and effectiveness.^21^ Thus, socially accountable training to understand the needs and willingness of providing care for those at highest risk is critical to ensuring that efforts at HIV detection, linkage to HIV care and retention are successful.

The extents to which MSM are stigmatized by health care providers and the attitudes of medical students towards MSM have been investigated in several contexts, yet to the best of our knowledge, no studies have focused on those issues in Haiti.^18^^,^^22^^,^^23^ The aims of this study were firstly, to assess the attitudes that medical students harbour towards MSM and better understand the extent to which stigma and other factors may impair actual and future healthcare, and secondly, to explore suggestions of opportunities for making such services socially accountable. We chose to focus on MSM because the social climate of stigma, fear of discrimination and lack of knowledge among health care providers are part of the reasons why this group is the most marginalized and still left behind by the health systems, putting them at increased risk of HIV.^24^

## Methods

### Study design

We utilized a qualitative study design by using a grounded theory approach regarding the context of Haiti.^25^ We sought to assess the attitude of medical students towards MSM living with HIV based on an inductive approach, which provided the framework for data analysis.^26^^,^^27^

### Study setting

Quisqueya University School of Health Sciences is a medical school located in Port-au-Prince, the capital city of Haiti. As the largest private university, the school of Health Sciences comprises 800 students originating from all administrative departments of the country. The six-year medical programme is divided into two-year basic sciences and four-year clinical sciences in partnership with associated academic hospitals located not only in the capital city but also in several rural and urban regions of the country ensuring practice through various cultures and contexts. The programme also entails clinical placements in infectious diseases and HIV settings where students work under the supervision of clinical tutors. As part of the social accountability reform process for accreditation renewal, efforts are being made to have a substantial basic science, clinical, and epidemiological community-oriented research activity within the university and associated academic hospitals.

### Participants and data collection

We used purposive sampling to select the research participants, which comprised 11 males and 11 females. Ages of the participants vary from 23 to 26 years; 20 of them identified themselves as Christians (Catholic and Protestants), and two did not mention their religion. The participants were in their final step of the clinical sciences programme and already spent three years in clinical placement at the associated academic hospitals. Among them, 17 indicated that they have already provided care for MSM during their clerkships in HIV facilities. For those who indicated that they had never served any MSM and therefore did not have any experience to share, we asked them to imagine what would happen if they were to take care of MSM. Eligibility was met if they were registered in the final year and had clinical training and practice in providing care to PLHIV. In order to ensure maximum variation, we managed to elicit views from diverse categories of students with different ages, sex, city of origin and religious beliefs.

The interview guide was made in accordance with the study objectives. The questions were open-ended, with probes used to explore points raised by interviewees or for clarification where more information was required. Data was collected through in-depth interviews from the 22 participants selected. We explored their personal views on PLHIV in general, their attitudes regarding MSM living with HIV, their willingness to provide care for them, their experiences with MSM in the clinical settings, and suggestions towards provision of sexual healthcare targeting and appropriate for MSM. The interview guide was available depending on the language preference of the interviewee (French or Haitian Creole). All participants preferred to be interviewed in Haitian Creole, but some responses were also provided in French; interviews lasted between 35 to 55 minutes. We allowed enough time with participants to ensure that adequate data were collected during the interview and continued the process until we reached saturation. Participants were free to ask questions on issues they felt were not clear to them. Permission was obtained to audio record the interviews. The interviewer was ready to take notes where an audio recording would have been declined. Ethical approval for the study was obtained from the Haitian Group for the Study of Kaposi's Sarcoma and Opportunistic Infections' human right committee and the Cornell University Weil Medical College's Research Ethics Board. Prior to interviewing, the study purpose and expectations of involvement were explained in Haitian Creole to the participants. We obtained oral and written informed consent from all participants. Data were stored in a secure place, and the anonymity was maintained through de-identification.

### Data analysis

Recorded interviews were transcribed verbatim. We reviewed the transcribed data to ensure understanding and then compared these transcripts with the original audio recordings for accuracy. The primary author read and reviewed the transcripts multiple times and selected one transcript for the initial open coding. To validate the coding process, a clean copy of the same transcript was de-identified and given to another experienced qualitative researcher to conduct a separate independent open coding which was later verified by co-authors. The primary author assessed both coding outputs and came up with one generic coding frame for indexing the rest of the codes. Relationships and comparisons between themes were generated from the coding frame in an iterative process. This ensured that attention was given for consistent patterns within the data focusing on similarities and differences on responses given by participants to aid analysis and interpretation. Our approach to data analysis was based on the thematic analysis in line with the study aim.

Four techniques were used to support the trustworthiness of the work: credibility, dependability, conformability and transferability.^28^^,^^29^ Prior to data collection and in order to understand the contextual factors relating to the HIV care continuum for MSM, three meetings and two participant observation sessions were held with MSM patients, HIV caregivers and medical students at two HIV-associated academic centres and at the main campus of the Université Quisqueya. Credibility was established by selecting in-depth interview method for the data collection and by the researcher who conducted the interviews being familiar with the context. Dependability was established by describing the data analysis in detail and providing direct citations to reveal the basis from which the analysis was conducted. The citations used in this article were translated from Haitian Creole into English with the help of a translator, to maintain accuracy and context as much as possible. The conformability and consistency of the analysis were established by holding meetings for the authors to discuss preliminary findings, emerging codes and themes until a consensus was reached. To enhance the transferability of the findings, a description of the context, selection and demographics of participants, data collection and process of analysis is provided to enable the reader determine whether the results of this study are transferable to another context.

## Results

The study findings have been grouped by themes. Thus, we present the themes and supporting quotations to illustrate the main findings.

Willingness to provide care to MSM infected with HIV

All students indicated that they were or would be comfortable serving MSM, although a few of them expressed some level of discomfort. They expressed their willingness to provide MSM with comprehensive HIV care. Their expressions were based on the right for MSM to receive care, the moral responsibility of healthcare professionals and the perceived health disparities regarding MSM in the population.

### Right to receive comprehensive care

The participants of the study stated that every human being has the right to receive adequate healthcare services regardless of their sexual practices and sexual orientation. They expressed their readiness to address MSM's medical issues unreservedly.

"…MSMs are human beings so they deserve and have the right to good treatment...we are the future of the health society in this country and we need to be ready to consider everyone as equal…" [Student 3, male, 23 years old]

### Moral responsibility

Participants indicated that in their capacity as students soon–to–be graduated, taking care of MSM is a moral duty in regard to the Hippocratic Oath.

"... We will have Hippocrates' oath and diploma ceremony so we'll have to respect our promise…" [Student 9, female, 23 years old]

Thus, they expressed their compliance to provide MSM with services in much the same way as they would for other patients and maintain the expected level of confidentiality.

"I don't think we have the choice; I choose to study medicine so I can take care of sick people... straight or gay, they need us…we are here to listen and to understand them…" [Student 16, male, 23 years old]

### Perception of health disparities

Stigma from healthcare professionals was identified by the participants as a reason why MSM may be reluctant to take up healthcare services. Participants agreed that this is one of the reasons why they have a responsibility to provide care without discrimination.

"…We cannot let them apart because they are already neglected by some doctors and nurses... I saw a nurse talking really bad to a boy because he was acting and talking like a girl …" [Student 13, female, 24 years old]

### HIV risk reduction

Some participants expressed their concerns about the high risk of HIV infection among MSM, who are perceived as a bridge through which HIV is spread to the general population. Thus, they expressed their willingness to take care of them in order to reduce this risk.

"…gay men have greater risk to get HIV and they have sex with girls too; that's like a [cycle]. If we let them out, the epidemic will become very important again…" [Student 16, male, 23 years old]

### Layered stigma and lack of specific skills to provide MSM with comprehensive HIV care

Evidence from studies suggests that MSM-related training reduces homophobic sentiments from health care providers.^30^ We asked the participants if their training has provided them with sexual health knowledge to address MSM specificities.

### Patient-provider misunderstanding

The findings revealed that medical education curriculum does not consider MSM specificities. Students explicitly expressed their limitations in relation to health of sexual minorities. Some of them explained that their professors and clinical instructors do not address MSM particularities and went further by explaining some stigmatizing attitudes they had noted from them.

"… I don't think we learn much because some professors laugh when we bring discussions about gay. They don't like talking about them; it's a sensitive topic…" [Student 8, male, 24 years old]

Although they all agreed that taking care of MSM is an obligation, some male participants expressed some level of discomfort.

"…I don't mind take care of them but they have to respect me. Gay people are too open. In the hospital they called me…told stories and touching me…I can't have contact with them…" [Student 10, male, 24 years old]

They affirmed that MSM could try to harass them during medical examination. Moreover, some students think that taking care of not only MSM but also any PLHIV require specific protection equipment like gloves and masks. These sentiments suggest a lack of medical and communication skills in HIV and sexual health.

"I called a nurse to assist me… so he doesn't touch me…sincerely I was afraid staying alone with him in the examination room…sometimes they are violent…" [Student 2, female, 24 years old]

### Layered stigma about homosexuality

Some students expressed a level of discomfort when it comes to holding discussions with MSM about sexual relationships.

"I would feel uncomfortable asking questions about their private life. I don't need to know their private life, it's disgusting. This is why they are sick…" [Student 14, male, 26 years old]

When we probed further about the feeling of discomfort, some of them expressed their disagreement of same-sex relationship and their disposition to try to convince MSM to leave such practice.

"…This is a sin. God doesn't like that, so as a doctor I can try to convince them to leave such life…" [Student 10, male, 24 years old]

These findings suggest the need to continue questioning their ability to properly address sexual health issues and confirm the need for medical curriculum to address MSM-sensitive topics.

### Implications for social accountability

Social accountability in health stems from a social contract which outlines the roles of training institutions in regard to the societies within which they are located and from where they draw their resources.^31^ A socially accountable physician is one who has a deep and profound understanding of his or her community responsibilities, having been trained in the community with a view towards population health and eliminating health inequities in partnership with community members.^32^

In regard to this principle, we wanted to explore what the students would suggest to address the gap related to MSM-specific sexual health in their medical education. They expressed key ideas on how to make it more inclusive and socially accountable through the following two areas:

### Inclusive medical education

Some students indicated the need for MSM-related issues and specificities in their curriculum.

"I think a training in MSM-related care will help us understand them better" [Student 18, female, 24 years old]

They suggested classes on physical and psychological needs of sexual minorities.

"…We see them everywhere but we don't even know how to properly communicate on issues that may affect their health…" [Student 16, male, 23 years old]

### Non-judgmental and caring attitudes of professors

One of the solutions that the students pointed out is a change in attitude of their professors who sometimes mock MSM during classes and clinical training even if they don't agree. They went further and called for a need to train their professors.

"…I think our professors don't give us a chance to learn about sexual minorities' health because they don't know much about them either. If they can participate in conferences about gay men, they would probably be more tolerant in their words and attitudes during classes…" [Student 8, male, 24 years old]

Understanding patients' needs with respect to their identity and preserving the human dignity of individuals with their multiple social identities are essential, and these findings reinforce the concept. Implications for social accountability in regards to specific health needs of MSM in Haiti stems particularly from two values: equity—which requires student learners to engage with minority groups, and quality—which promotes multi-professional and policy partners in teamwork to address major social determinants of health.^33^

What then do these findings imply for medical education in Haiti? It can enable a reflection process for medical education policymakers and healthcare professionals to analyze medical curricula, legislation and public health policies more critically while considering contextual factors which can perpetuate the cycle of social disparities. It can also help to better understand and address the social health inequalities in regard to social determinants of health. Concretely, applying the social accountability values through interventions will give visibility to stigmatized and marginalized populations such as MSM and improve the competencies of healthcare professionals to adapt their practices to the complexity of population needs and health system stakes.^34^^,^^35^

## Discussion

Our study revealed three main findings: a) participants expressed their willingness to provide MSM with comprehensive healthcare; b) medical education curriculum does not consider MSM-specific sexual health and emerging layered stigma attitudes while providing comprehensive services through a discussion about same-sex relationship; and c) suggestions of a more inclusive medical education and non-judgmental, caring attitudes of professors and clinical instructors.

In this study, the majority of students were in support of adequate healthcare delivery to MSM infected with HIV. They stated that MSM has health needs like other patients and deserve access to appropriate healthcare. They reflected on their professional responsibility by referring to the Hippocratic Oath to highlight their willingness. Our findings are in line with another study conducted in Malawi, showing that medical students expressed their entire willingness to provide MSM with appropriate healthcare.^36^ However, in contrast, negative attitudes towards MSM were found to be high in Malaysia and Russia.^17^ Access to healthcare in some countries where MSM are not protected by law is still challenging. However, it has been proved that negative attitudes of health care providers have shifted due to the implementation of educational interventions; one of the recommendations made by the participants.^37^

Another reason for their reflection was to reduce health inequity and global HIV risk. This finding refers to the recommendations by the World Health Organization for MSM.38 Stigma is indeed perceived and experienced in healthcare settings, and anxiety about HIV status, its social consequences and confidentiality concerns are major deterrents to accessing HIV care and exacerbate health inequity among MSM. A study from Ghana supports the contribution of tailored interventions to reduce health inequity.^39^ Moreover, another study from the United States demonstrated that scaling up a group of interventions targeting both health care providers and MSM like sensitization sessions and peer support are important.^40^

While students feel the obligation to provide care to MSM like any other patient, some of them would not be comfortable. Homophobia represents a major barrier to accessing HIV services among MSM.^12^ They also have limited access to specialized programmes, even in comparison with people who inject drugs and sex workers.^41^ While considering the fact that MSM may opt not to reveal their sexual orientation to health care providers due to these attitudes, more studies are needed to understand all forms of homophobia in clinical settings. Moreover, programmes focused on clinical interventions need to also consider human right issues.

The students clearly indicated that they did not have the requisite skills necessary to handle MSM health specificities; most of them felt that they not only needed to be clinically trained, effective communication training sessions required to be delivered to their professors. Several previous studies have documented that a lack of specific skills can affect continuum of care for MSM.^36^^,^^37^^,^^40^  In contrast, other studies showed that health care providers trained in MSM specificity provide better holistic care while considering the social aspects of their lives.^30^ It follows that training of health care providers will lead to skill improvement for quality of healthcare; however, efforts to shift attitudes have to consider the cultural components of the society.

By mentioning the negative attitudes of their professors, some students pointed out their role in shaping the actual and future model of care in Haitian society. In 1990, a study from the United States found that medical school professors can be a source of negativity regarding HIV positive and marginalized groups.^42^ In fact, the senior doctors define and portray attitudes during the learning process, which will probably stay with future healthcare professionals throughout their career. However, participatory training methods and interventions tested by other studies showed valuable tools for changing attitudes and decreasing biases.^30^

Although the issues that MSM face are documented, gaps remain in the medical curriculum. Some countries have started incorporating MSM health in their medical curriculum, while Haiti has limited engagement with the topic. Medical students need didactic sessions to be incorporated into the curriculum so that their knowledge of how social determinants impact the health of MSM can be addressed alongside interventions and research efforts on increasing comfort and mitigating stigma.^31^^,^^33^^,^^43^

Evidence of the link between health training institutions and communities has been around for a long time; however, it is only in recent decades that social accountability of health education has become more formalized.^31^ The "social contract" defines the duties of the training institutions with regard to all aspects of the societies in which they are established and where they draw their resources.^32^^,^^33^ Thus, solutions and strategies that gradually stimulate the adaptation of health professions to the realities of every community, including MSM, are necessary to strengthen professional ethics for the respect of rights, duties and freedom without stigma.

This is a qualitative study that explored attitudes of medical students towards MSM living with HIV, an area clearly lacking in research. The results represent the views of a single medical student population in Haiti to a single stigmatized group; this may limit the generalisability of the study findings. However, we tried to improve external validity by selecting participants from different backgrounds and culture. Our study did not evaluate a respondent's level of practice during medical education to determine whether attitudes changed with an increased amount of experience. However, the description of acceptability to provide HIV-related health services among students as elicited in this study could be valid in another setting with similar context.  Despite these limitations, participants came from different backgrounds in terms of religion and city of origin; they also practice in the same academic hospitals with students from other medical schools and share same professors and clinical instructors. By documenting the attitudes, our study provides critical insight into the extent to which stigma towards MSM living with HIV is endorsed—not only by the students but also by the professors—and highlights the need for stigma mitigation interventions.

## Conclusions

This qualitative study explored the attitudes of medical students towards MSM living with HIV in Haiti. The study showed that medical students were willing to provide MSM-focused HIV care services. They recognized the right for MSM to have equal access to services as other patients and proposed intervention strategies to address MSM-specific gaps in their medical education. However, future researches are required to capture more views, experiences and propositions from not only medical students but experienced healthcare givers in order to propose socially accountable interventions targeting revision of curriculum and more equitable HIV care for MSM in Haïti. The shift from evidence to action requires synergy in the socio-centric evolution of health to enhance relevance, quality, efficiency and equity of the social accountability of medical education, as stigmatized populations and sexual minorities can no longer be kept unchecked in the era of 'Leave no one behind' for a world free of HIV.

### Authors' contributions

WD, CA, CR, JWP and YC conceived the original research idea and led the design of the study. WD, CA and YC developed the protocol and the topic guide. WD, CA and YC conducted the analysis. WD and CA developed the first draft of the article. All authors oversaw the development of the article and contributed to the revisions. All authors reviewed and approved the final draft.

### Acknowledgements

We thank the Université Quisqueya's administration and the Dean's office for their support and assistance and all the students for their willingness to participate in the study.

### Conflicts of Interest

The authors declare that they have no conflicts of interest.
